# Coronary Artery Bypass Grafting in Patients with Chronic Kidney Disease: Chronic Kidney Disease Has an Independent Adverse Effect on the Long-Term Outcome of Coronary Artery Bypass Grafting

**DOI:** 10.1155/2022/4994970

**Published:** 2022-04-26

**Authors:** Daisuke Endo, Taira Yamamoto, Kan Kajimoto, Satoshi Matsushita, Shizuyuki Dohi, Akie Shimada, Yasutaka Yokoyama, Hiroaki Io, Yusuke Suzuki, Minoru Tabata, Atsushi Amano

**Affiliations:** ^1^Department of Cardiovascular Surgery, Juntendo University, 2-1-1 Hongo, Bunkyo-ku, Tokyo 113-8421, Japan; ^2^Department of Cardiovascular Surgery, Juntendo University Nerima Hospital, 3-1-10, Nerima-ku, Tokyo 177-8521, Japan; ^3^Department of Cardiovascular Surgery, Juntendo University Shizuoka Hospital, 1129 Nagaoka, Izunokuni, Shizuoka 410-2295, Japan; ^4^Department of Nephrology and Hypertension, Juntendo University, 2-1-1 Hongo, Bunkyo-ku, Tokyo 113-8421, Japan

## Abstract

We examined short- and long-term outcomes of coronary artery bypass grafting (CABG) in patients with ischemic heart disease and the effect of renal function on these outcomes. We included 2783 patients who underwent primary elective CABG at a single institution between 2002 and 2020 (age: 67.6 ± 10.2 years; male: 2281 male). They were stratified based on their preoperative estimated glomerular filtration rate and underwent off-pump CABG (completion rate, 98.1%); 57.6% cases used bilateral internal thoracic arteries (BITA). In-hospital mortality rate was 1.0%. Logistic regression analysis revealed that low left ventricular function (<40%), but not chronic kidney disease (CKD) severity, was an independent predictive risk factor for postoperative hospital mortality. Significant differences existed in respiratory complications, infections, and hospitalization duration according to CKD severity. Deep sternal wound infection rate was 0.5%. The mean follow-up period was 7.1 (0–18.5) years. Estimated 10-year survival rates were negatively correlated with CKD severity; in the Cox hazard model, severe CKD was an independent predictor of long-term survival. We examined the relationship between preoperative and intraoperative factors and their effects on long-term survival using propensity score matching by dividing the renal function severity into G1–2 and G3–5. Renal function severity, age, and operative time were independent risk factors. No prognostic improvement was observed with BITA grafts; graft patency was superior in the right internal thoracic artery (52/52; 100%) than in the great saphenous vein (48/59; 81.4%) in G5. Post-CABG in-hospital mortality was unrelated to renal function, but CKD severity strongly influenced long-term survival. Operation time was an important predictor of long-term prognosis in patients with impaired renal function. Treatment plans, including graft and anastomosis-site selections, should be designed to shorten the operation time. In conclusion, using the right internal thoracic artery in CABG is more beneficial in patients with CKD and cardiovascular comorbidities.

## 1. Introduction

The prevalence of cardiovascular disease in patients with chronic kidney disease (CKD) is twice that in patients with normal kidney function [1]. In a study on adult CKD patients who were not receiving dialysis or renal transplantation, Go et al. reported a negative correlation of the adjusted hazard ratio for cardiovascular events with the estimated glomerular filtration rate (eGFR). There are also independent stepwise associations between lower eGFR, risk of cardiovascular mortality, and incidence events with in-hospital treatments in patients other than those undergoing dialysis [2].

Renal glomerular filtration function decreases at an approximate basal rate and a generally linear manner because of the accumulation of metabolic, hemodynamic, dietary, and other stresses [3]. However, there are cases of rapid decline in eGFR. Regardless of the original renal function, patients with extremely low eGFR are likely to have diabetes or hypertension and are also likely to be chronic smokers. A rapid decline in eGFR is strongly associated with mortality and combined cardiovascular disease events, regardless of the baseline eGFR [4].

Patients with comorbid renal dysfunction have an increased risk of cardiovascular and cerebrovascular ischemic events due to systemic inflammatory activation, dysfunction of vascular endothelial cells, atherosclerosis, and promotion of thrombus formation [2, 5, 6]. Furthermore, patients with renal dysfunction who have undergone coronary artery bypass grafting (CABG) are at risk of postoperative acute renal failure and related complications, which affect their short-term outcomes and long-term prognosis after CABG [7]. Moreover, percutaneous coronary intervention (PCI) is associated with the risk of acute renal failure due to the use of contrast media and atheroembolism, which could contribute to the reduced probability of revascularization among patients with ischemic heart disease than among those with normal renal function [8, 9].

In Japan, the number of patients requiring hemodialysis is increasing, with 340,000 patients reported to have required hemodialysis in 2018. Approximately, 50% of deaths among patients undergoing maintenance dialysis results from heart diseases, including heart failure and myocardial infarction [10]. There has been no significant change in the long-term outcomes of CABG in patients undergoing dialysis; moreover, the use of the bilateral internal thoracic arteries (BITA) has not been suggested to improve patients' outcomes [11]. Additionally, numerous studies have shown that the perioperative and long-term outcomes of CABG cannot be improved with the increasing severity of CKD and hemodialysis [12–14]. A previous study considered off-pump CABG (OPCAB) as appropriate in such cases [15]; however, another study described the many changes in surgical procedures during operation and lack of clarity regarding the graft to be used and surgical strategy [16].

This study was aimed at evaluating the relationship of preoperative CKD severity with perioperative and long-term outcomes, as well as the efficacy of bypass grafts in patients who have undergone CABG.

## 2. Materials and Methods

### 2.1. Patients

Between July 2002 and January 2021, we performed CABG in 3720 consecutive patients at Juntendo University Hospital. Among them, we excluded patients who underwent reoperation or combined operations, including valvular or aortic surgery. Finally, we included 2783 patients who underwent primary isolated CABG (mean age: 67.6 ± 10.2 years; 2281 [81.9%] male individuals). This retrospective study analyzed the operative outcomes of CABG and long-term follow-up examination findings in 2783 patients with different renal functions.

### 2.2. Surgical Procedure

Generally, OPCAB was planned for all patients. We used cardiopulmonary bypass for patients with severe heart decompensation or low left ventricular ejection fraction (LVEF). We used arterial grafts, which were mainly the internal thoracic artery (ITA), right gastroepiploic artery (GEA), or radial artery (RA). These grafts were harvested through the skeletonization method using an ultrasonic scalpel (Harmonic Scalpel; Ethicon Endosurgery, Cincinnati, OH, USA). The great saphenous vein (SVG) was harvested using the conventional method. We evaluated the graft function using a flow-trace recording of transit time after completing each graft and just before chest closure. In our institution, patients on hemodialysis or those with reduced urine output during surgery are made to undergo continuous hemodiafiltration (CHDF) in the intensive care unit. Afterward, patients undergoing dialysis were transferred to the hemodialysis unit after achieving hemodynamic stability. Nafamostat mesylate was used as the anticoagulant during CHDF.

### 2.3. Graft Design

Waitlist patients underwent three-dimensional computed tomography (3DCT) to evaluate most grafts. The most important strategy for our myocardial revascularization was in situ grafting of the left internal thoracic artery (LITA) into the left anterior descending artery. We considered grafting of the right internal thoracic artery (RITA) into the left circumflex artery territory (LCX); further, we used the SVG or RA when the graft length or other anatomical factors rendered the RITA unsuitable. For patients with severe renal dysfunction, the RA was often left to provide vascular access for future hemodialysis. Contraindications for use included anatomical factors, such as the end of the circumflex branch being insufficiently long for the RITA and stenosis < 90% in the LCX region. We used the RITA if the aforementioned conditions were satisfied even in patients with obesity, diabetes, and advanced renal dysfunction. The GEA was used for the right coronary artery (RCA) in cases with severe stenosis of >90% of the target vessel. However, severe calcification and stenosis of the celiac artery and GEA were limitations in patients with severe renal dysfunction, which discouraged the use of the GEA. We used a partial clamping procedure for the proximal anastomosis of the ascending aorta. In contrast, we used Enclose II (Novare Surgical Systems, Cupertino, CA, USA) or PAS-Port automated proximal anastomosis (Cardica, Inc., Redwood City, CA, USA) for patients with unfavorable ascending aorta disease.

### 2.4. Management of Infection Control and Wound Healing

There are significant challenges regarding surgical site infection and wound healing among immunocompromised patients because of severe renal dysfunction or severe nephrotic syndrome. There have been several changes over the years, but our current multidisciplinary protocol, which has been in place since September 2009, includes the following: (1) preoperative screening for methicillin-resistant *Staphylococcus aureus* (MRSA), and intranasal administration of mupirocin ointment for MRSA carriers; (2) administration of antibiotics (cefazolin administration 30 min before skin incision, readministered intraoperatively at 3 h intervals, and early antibiotic discontinuation postoperatively; however, MRSA carriers receive either vancomycin hydrochloride or arbekacin sulfate); (3) wound cleaning; (4) wound closure through the placement of JVAC/blake drain 10 Fr (ETHICON, New Brunswick, NJ, USA) as a subcutaneous drain for 4 postoperative days; wound dressing by placing DuoACTIVE plus Aquacel Ag. (ConvaTec Group Plc, Berkshire, UK) for 5 days; (5) thorough perioperative serum glucose control (<180 mg/dL); (6) preoperative albumin correction (if serum albumin < 3.0 mg/dL); and (7) postoperative albumin correction (if serum albumin < 3.0 mg/dL).

### 2.5. Data Collection

We obtained informed consent from all patients preoperatively to use their medical records in this study. Additionally, all the patients provided written permission to publish this manuscript. This study was approved by the Clinical Ethics Committee of Juntendo University Hospital (approval number: NO. E21-0159-H01 [October 28, 2021]) and was conducted in accordance with the principles of the Declaration of Helsinki. We collected the following data: preoperative treatment history, preoperative and postoperative general condition, medical treatment, biochemical tests, transthoracic echocardiography, cardiac catheterization (CAG), and graft evaluation through electrocardiography (ECG) gated three-dimensional computer tomography (3D-CT). We obtained data regarding the long-term prognosis from the medical records of Juntendo University Hospital.

### 2.6. Definition of CKD

Patients were divided into five categories of preoperative CKD severity based on their eGFR. We used the following Chronic Kidney Disease Epidemiology Collaboration renal function estimation formula, which is strongly related to post-CABG outcomes: for women with a plasma creatinine ≤ 0.7 mg/dL: (plasma creatinine/0.7)^−0.329^ × (0.993)^age^ (×166 if black, ×144 if white or other); for women with a plasma creatinine > 0.7: (plasma creatinine/0.7)^−1.209^ × (0.993)^age^ (×166 if black, ×144 if white or other); for men with a plasma creatinine ≤ 0.9 mg/dL: (plasma creatinine/0.9)^−0.411^ × (0.993)^age^(×163 if black; ×141 if white or other); and for men with plasma creatinine > 0.9: (plasma creatinine/0.9)^−1.209^ × (0.993)^age^ (×166 if black; ×144 if white or other) [17, 18].

CKD severity was classified as follows: G1, normal (eGFR ≥ 90 mL/min/1.73 m^2^); G2, mild CKD (eGFR ≥ 60, <90 mL/min/1.73 m^2^); G3, moderate CKD (eGFR ≥ 30, <60 mL/min/1.73 m^2^); G4, severe CKD not requiring chronic hemodialysis (eGFR ≥ 15, <30 mL/min/1.73 m^2^); and G5, hemodialysis dependent or most severe condition (eGFR < 15 mL/min/1.73 m^2^).

### 2.7. Endpoints and Definitions

The study's primary endpoints were short-term operative mortality and remote major adverse cardiovascular and cerebrovascular event (MACCE) composite measure of all-cause mortality, myocardial infarction, and stroke. The secondary outcome was perioperative complications. As a subanalysis, we classified the patients into the G1–2 and G3–5 groups and examined the involvement of preoperative and intraoperative factors in the patients' long-term survival using propensity score matching (PSM). We also examined the impact of the graft used on the survival and patency rates. Diabetes mellitus was defined as diabetes mellitus diagnosed and managed using diet, oral agents, and insulin therapy. Diabetic nephropathy was defined as proteinuria or decreased renal function (eGFR < 60 mL/min/1.73m^2^) persisting for >3 months in patients with diabetes mellitus, and complications of diabetic retinopathy were also considered as diabetic nephropathy [19]. Hypertension was defined as currently undergoing medical treatment or having a documented systolic or diastolic blood pressure of ≥140 or ≥90 mmHg, respectively. Dyslipidemia was described as a preoperative low-density lipoprotein cholesterol level of ≥140 mg/dL or currently undergoing medical treatment. Peripheral arterial disease was defined as previous treatment or severe stenosis (>75% stenosis) of peripheral vessels, including the abdominal aorta. Postoperative stroke was described as a new onset of paralytic symptoms in the central nervous system. Deep sternal wound infection (DSWI) was defined as a chest wound infection involving sternal or mediastinal tissue during the follow-up period of the study. The secondary outcome was the patency of the bypass graft on CAG and ECG-gated 3D-CT during the postoperative and long-term periods.

### 2.8. Statistical Analyses

The normality of the distribution of continuous variables was assessed using the D'Agostino-Pearson analysis. Descriptive statistics were used to describe the study participants. Continuous variables are presented as means and standard deviations, while categorical variables are presented as total numbers and percentages. Results regarding initial events and possible long-term outcomes are presented as numbers and percentages. Continuous variables were compared using the *t*-test, Wilcoxon test, and Mann–Whitney *U* test (nonparametric variables). Categorical variables were compared using Pearson's v2 test. The survival rates for all-cause and cardiac-related deaths were evaluated using the Kaplan–Meier method. Statistical significance was established using the log-rank test. Univariate and multivariate logistic regression analyses were used to identify independent predictors of hospital mortality. Additionally, univariate and multivariate Cox proportional hazard regression analyses were used to analyze all-cause mortality during the follow-up period. In this study, PSM analysis was used to analyze the correlation between the long-term survival of patients in the G1–G2 group and that of those in the G3–5 group to reduce potential selection bias. The nearest available matching method was used for PSM with a caliper of 0.03. The level of significance was set at *P* < 0.05 with a two-tailed test. All statistical analyses were performed using the JMP software version 16.0 (SAS Institute, Cary, NC, USA).

## 3. Results

### 3.1. Demographics and Baseline Characteristics

Table 1 shows the background characteristics of the patients. There were 256 (91.4%) dialysis patients in the G5 group. The patients in the G3 group were significantly older than those in the other groups; however, there was no significant age difference between patients with G1–G2 and those with G5. Hyperlipidemia was most common in G1 and decreased with worsening renal function. The prevalence of diabetes mellitus (especially patients on insulin) was positively correlated with CKD severity. The prevalence of hypertension, peripheral atherosclerotic disease, and stroke was positively correlated with CKD severity. Additionally, LVEF was negatively correlated with CKD severity, while the complication rates of mitral regurgitation and atrial fibrillation were positively correlated with CKD severity; however, there was no significant difference in the New York Heart Association classification. CKD severity was negatively and positively correlated with hemoglobin (due to renal anemia) and C-reactive protein (CRP) levels, respectively. Overall, the predictive risk score was positively correlated with CKD severity (Table 1).

### 3.2. Operative Results and Outcome In-hospital Stay

Table 2 shows the patients' intraoperative characteristics and early results. There were high rates of OPCAB (>96%) in all groups. The BITA grafts were used in 62.3% of all patients. The average number of anastomoses was >3.4 and 3.1 in G1–G4 and G5, respectively. There were no significant among-group differences, with 55–60% of patients in each group receiving BITA grafts. The use of other arterial grafts, including the GEA, was lower in G5, especially in patients undergoing hemodialysis. As an alternative, the use of the SVG was positively correlated with the severity of CKD. The mean operative time was 280 min, which was shorter in the G5 group with fewer anastomoses; moreover, the operative time was not prolonged due to CKD severity. In all groups, there was good postoperative reexploration at <1%. Additionally, the frequency of using intra-aortic balloon pumping was approximately 5%, with no significant among-group differences. The frequency of postoperative respiratory complications and infections was positively correlated with the severity of CKD. However, the incidence of DSWI was approximately 1% in each group. The incidence of postoperative blood transfusion and atrial fibrillation was positively correlated with CKD severity. The duration of hospitalization was positively correlated with the severity of CKD given the increase in complications. The hospital mortality rate was lower than the predicted risk score for G1–G4 (<1%) but almost similar to the predicted risk score for G5 (4.7%).

Table 3 shows the results of multivariate logistic regression analysis for hospital mortality. Preoperative CKD severity was not an independent risk factor for postoperative hospital mortality after CABG; however, low LVEF (<40%) was an independent risk factor for hospital mortality.

### 3.3. Prognosis during Follow-Up

**Figure 1:**
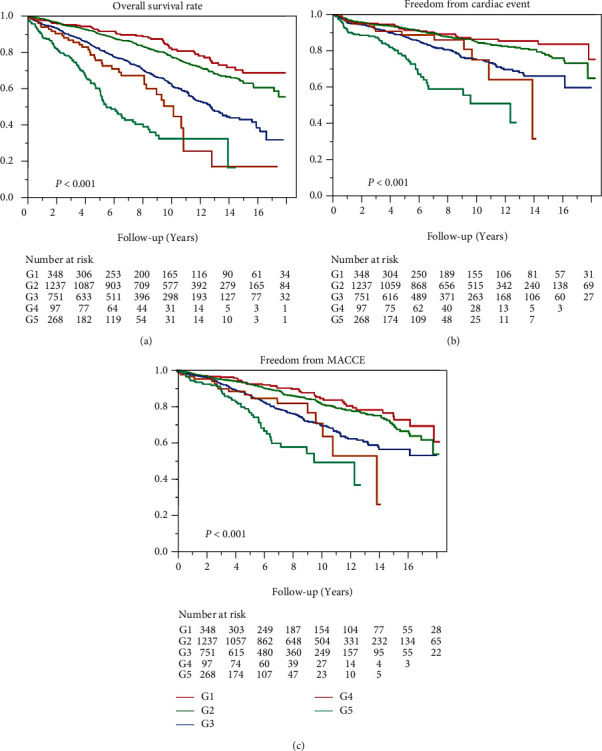
Overall survival rate (a), freedom from cardiac events (b), and major adverse cerebrocardiovascular events according to the severity of chronic kidney disease.

The follow-up rate was 96.0%, with a mean follow-up duration of 7.1 ± 4.8 years (range, 0–18.5 years). Overall survival (Figure 1(a)), freedom from cardiac accidents (Figure 1(b)), and MACCEs (Figure 1(c)) were negatively correlated with CKD severity.

The overall 5- and 10-year survival rates were 94.8% and 91.2% in G1, 90.5% and 78.1% in G2, 79.1 and 59.4% in G3, 73.3% and 51.5% in G4, and 60.2% and 32.7% in G5 (*P* < 0.0001), respectively. There was a negative correlation of long-term survival with the CKD severity. The 5- and 10-year rates of freedom from cardiac accidents were 93.9% and 91.0% in G1, 93.0% and 85.4% in G2, 90.0% and 76.5% in G3, 87.9% and 75.0% in G4, and 81.2% and 53.3% in G5 (*P* < 0.0001), respectively. The 5- and 10-year rates of freedom from MACCEs were 93.1% and 90.2% in G1, 91.1% and 80.5% in G2, 85.4% and 68.0% in G3, 81.3% and 68.6% in G4, and 75.9% and 47.3% in G5 (*P* = 0.001), respectively.

Table 4 presents a summary of the results of the Cox hazard model. Preoperative CKD severity was an independent risk factor for long-term mortality (G1/G4: odds ratio [OR], 2.82, 95% confidence interval [CI], 1.27–6.24; G1/G5: OR, 5.78, 95% CI, 2.76–12.09), together with diabetes mellitus, peripheral vascular disease, previous stroke, mitral regurgitation > moderate, preoperative atrial fibrillation, and low left ventricular function. Contrastingly, BITA grafting was a strong protective factor against all-cause mortality (hazard ratio, CI 0.65–0.90) (Table 4).

### 3.4. Subgroup Analysis

Table 5 shows the baseline characteristics of the patients. Based on the PSM performed on the data, the two groups (G1–2 and G3–5) have equal number of patients (909 patients in each group). There were significantly more male patients (84.8%) in the G3–5 than in the G1–2 group (73%; *P* < 0.001). Patients in the G3–5 group were not significantly older than those in G1-2 groups (*P* = 0.862). The result also revealed that there was more significant survival in the G1–2 groups (97.4%) compared with the G3–5 (91.5%) group (*P* < 0.001). Further, there were significantly more patients with prior cerebrovascular accident (history of stroke or transient ischemic attack) in the G1–2 groups (23%) than those in the G3–5 groups (14.5%). Further, the results showed significant differences in the follow-up period (*P* < 0.001), perivascular disease (*P* = 0.019), and mitral regurgitation (moderate or severe) (*P* < 0.001). However, there was no significant difference between the two groups in hyperlipidemia, hypertension, prior myocardial infarction, emergency, number of diseased vessels, LVEF, number of bypasses, and operation time.

Table 6 shows the multivariate coz regression analysis results for mortality in the follow-up. Based on our findings, out of all potential predictors tested for at the univariate level, only the operative time (*P* < 0.001) can significantly predict the mortality rate in the follow-up period. However, further analysis at the multivariate level revealed that sex (*P* = 0.047), age, and operation time (both *P* < 0.001) can significantly predict the mortality rate within the groups.

Table 7 shows the graft patency rates. The LITA showed a reasonable patency rate (>90%) in the short- and long-term follow-ups, regardless of CKD severity. However, the graft patency rate of SVG was negatively correlated with CKD severity. Contrastingly, the RITA showed excellent long-term patency even in the G5 group (Table 7).

**Figure 2:**
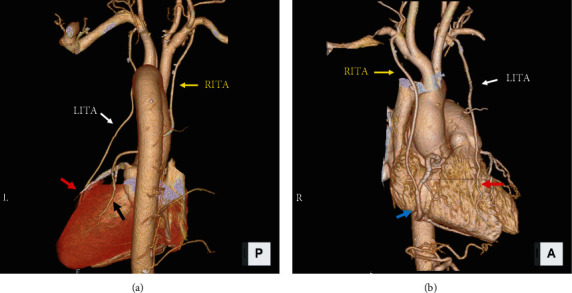
Postoperative Graft Imaging. (a) A 64-year-old woman with a 4-year history of hemodialysis due to nephrosclerosis underwent coronary artery bypass grafting for the circumflex artery using the in -situ right internal thoracic artery. Computed tomography angiography performed at 3 years postoperatively showed excellent graft patency. LITA: left internal thoracic artery (white arrow, (a)); LAD: left anterior descending artery (red arrow, (a)); RITA: right internal thoracic artery (yellow arrow, (a)); and RCA: obtuse marginal branch in the left circumflex branch (black arrow, (a)). (b) A 64-year-old woman with a 10-year history of hemodialysis due to diabetic nephropathy had undergone CABG surgery 1 year prior. Her ECG-gated 3D-CT findings were used to visualize the good patent grafts. LITA: left internal thoracic artery (white arrow, (b)); LAD: left anterior descending artery (red arrow, (b)); RITA: right internal thoracic artery (yellow arrow, (b)); and RCA: right coronary artery (blue arrow, (b)).

Postoperative ECG-gated 3DCT is presented in patients on G5 dialysis with good patency in both cases of bypassing the rotator cuff of the RITA and bypassing the RCA (Figure 2).

## 4. Discussion

This study was aimed at analyzing the short- and long-term outcomes of patients with ischemic heart disease undergoing CABG, correlated to CKD severity. We did not use cardiopulmonary bypass, which negatively affects renal function in most cases; the patients usually underwent OPCAB with a completion rate of 98.1%. Our study reported several significant findings. First, the in-hospital mortality rate was 1.0%. Multivariate analysis revealed that poor LVEF (<40%), but not CKD severity, was an independent predictive risk factor for postoperative in-hospital mortality. There were significant differences in respiratory complications, infections, and hospitalization duration according to CKD severity, although the deep sternal wound infection rate was 0.5%. Second, short-term outcomes, such as post CABG hospital mortality, were not related to renal function, although the severity of CKD had a substantial impact on long-term outcomes. Third, we performed PSM for the G1–2 and G3–5 groups separately, and this revealed that age and operation time were related to long-term survival in addition to renal function. Although the graft patency of BITA was superior, it did not improve the long-term outcomes in the G3–G5 groups.

Patients that require surgical and interventional coronary revascularization often present with CKD as an underlying medical condition. In a large cohort study using the STS National Database, Cooper et al. reported that 51%, 24%, and 2% of patients undergoing CABG presented with mild, moderate, and severe CKD, respectively, while 1.5% of the patients was undergoing hemodialysis [20]. Japan has a unique treatment pattern of continued maintenance dialysis. The Kyoto PCI/CABG Registry Cohort-3 reported that among patients with CKD, excluding dialysis, 4.8% and 6.8% of patients with an eGFR < 30 mL/min/1.73 m^2^ were treated with PCI and CABG, respectively, while 7.8% and 8.1% of patients undergoing hemodialysis were treated with PCI and CABG, respectively [21]. Among 4464 patients who underwent CABG, the Japan Adult Cardiovascular Surgery Database reported that 15.3% and 9.2% of the patients had serum creatinine levels > 2.0 mg/dL and were on dialysis, respectively [22]. In our study, the prevalence of G3, G4, and G5 were 31%, 3.7%, and 10.0%, respectively. This indicates that treatment for CKD complications is common in Japan and complex lesions in coronary arteries are also common. Furthermore, many physicians elect to perform CABG to avoid contrast-induced nephropathy and repeating interventions.

CKD severity is positively correlated with the proportion of diabetic cases. Although renal function generally worsens with age, severe CKD (G4–G5) was not predominantly observed in older patients. This could be attributed to the influence of diabetic nephropathy from a relatively young age. Additionally, CKD severity is positively correlated with the severity of anemia and CRP levels. This suggests that elevated CRP levels promote systemic inflammation and atherosclerosis. We found that renal dysfunction was associated with a higher prevalence of peripheral arterial disease and a higher rate of preoperative stroke complications. Additionally, CKD severity was positively and negatively correlated with a history of PCI and intraoperative left ventricle function, respectively. This indicates that in clinical practice, cardiac surgeons are more concerned about the surgical procedure and perioperative management of patients with more severe renal dysfunction.

Regarding postoperative complications, there was no significant correlation of the preoperative CKD severity with major perioperative cardiovascular events, including cerebrovascular accidents and postoperative heart failure. Numerous studies have reported a positive correlation of cerebrovascular complications with CKD severity; moreover, CABG involves a higher rate of cerebral complications than PCI [23, 24]. Our findings of a low rate of cerebral infarction could be attributed to the effect of OPCAB, which has been previously reported [25]. Additionally, the rate of reexploration associated with bleeding was 0–1% in each group, which was favorable without significant among-group differences. However, the duration of ventilatory management was positively correlated with CKD severity given the metabolic complications of anesthetics and inflammatory substances. Additionally, patients who required CHDF had more extended bed rest periods and increased respiratory complications due to atelectasis and pneumonia. The infection risk was strongly associated with CKD severity. Notably, the incidence of infections was approximately 1%, 2–3%, and 5% in the G1–G2, G3–G4, and G5 groups, respectively. Patients in the G4 group, who had not yet progressed to chronic dialysis, were more likely to postoperatively develop acute renal failure and required renal replacement therapy in the intensive care unit, which increased the number of cases that involved long-term ventilation. These interventions lead to an increase in the rate of infections. Additionally, anemia is a significant factor that affects early postoperative outcomes in a condition that is collectively termed “cardiorenal anemia syndrome” [26]. Anemia and CKD are correlated with worsening heart failure, especially in patients with diabetic nephropathy, where anemia worsens hospital mortality as well as causes postoperative heart failure and other complications [27].

In our study, the rate of deep sternal wound infection was favorable (approximately 1%) in all groups. Generally, the BITA was mostly used for grafting. The RA could not be used in patients with severe renal dysfunction, and the GEA could not be used due to abdominal artery calcification. Additionally, the SVG of the lower extremity may not have been suitable for grafting, as patients may have developed stasis dermatitis and phlebitis due to fluid overload, and anemia and hypoproteinemia due to worsening renal function. However, the BITA could be used in patients with extremely severe CKD. In our study, despite the use of the BITA, the incidence of wound infection remained low at 1% in all groups. We used the skeletonized harvesting technique, which removes most of the tissue surrounding the LITA. This technique extends the length of the harvested graft by 2–3 cm and increases the number of anastomotic sites, which allows for various graft designs [28]. Additionally, we performed negative-pressure sternal wound closure using a subcutaneous closed drain tube for 4 days [29]. This could have facilitated the prevention of surgical site infection, as previously reported. The Consultant Medical Diabetic Team strictly managed diabetes, with target blood glucose levels of 80–160 mg/dL [30].

Multivariate analysis did not reveal a correlation between in-hospital mortality and CKD severity. Specifically, the in-hospital mortality rate in the G1–G4 group was better than expected (1%). However, in the G5 group, which included patients undergoing hemodialysis, the in-hospital mortality rate was 4.7%. Patients with G5 who required hemodialysis had diabetes mellitus nephropathy with advanced systemic atherosclerosis and chronic glomerulonephritis for >10 years after starting dialysis. These cases could have contributed to postoperative complications and hospital mortality (21-25).

CKD is a risk factor for cardiovascular disease because it affects systemic vascular systems; moreover, cardiac dysfunction may exacerbate CKD. Severe CKD is associated with higher long-term morbidity and mortality rates. In this study, CKD severity was strongly associated with cardiac death, all-cause mortality, and incident stroke concerning long-term outcomes. We believe that long-term stabilization of cardiac function by CABG may prevent further deterioration of renal function.

Multivariate analysis revealed numerous risk factors for long-term mortality, with G5 being a particularly potent risk factor. Most patients with G5 were undergoing dialysis. Nevertheless, many patients with diabetes mellitus nephropathy and chronic glomerulonephritis are expected to have poor long-term survival given the likelihood of comorbidity with severe atherosclerotic disease of the carotid and peripheral arteries after ≥10 years. In our PSM subanalysis, age and surgery time were also independent risk factors for long-term survival, in addition to CKD severity (G3–5). Older people with chronic kidney disease are expected to have early deterioration of renal function when exposed to trauma, surgery, or drug stress. Furthermore, in cases of chronic kidney disease, CABG is often performed in patients with complicated coronary artery lesions. Moreover, a long operation time results in a more remarkable deterioration of renal function. Rosenfeld et al. reported that changes in the preoperatively proposed surgical plan or graft modifications were made in 39% more patients with CKD compared with the proportion of patients with normal renal function [16]. This further shows that patients with CKD or end-stage renal disease have more complicated and diffuse coronary artery lesions, which impede accurate preoperative surgical planning. Moreover, these patients often present intraoperatively with unexpected lesions and problems with anastomotic structures.

Cooper et al. reported that ITAs decreased surgical mortality in patients with severe CKD, regardless of their hemodialysis status [20]. Additionally, Ohira et al. found that the BITA graft was an independent protective factor for survival rate in the whole-cohort and subgroup analyses [13]. The efficacy of BITA grafts in patients with CKD is a concern when managing patients with severe renal dysfunction [31, 32]. In our study, the BITA graft showed favorable patency, even in patients with diabetes mellitus who had an eGFR < 30 mL/min/m^2^. Nakayama et al. reported an association between the use of BITA grafts and improved long-term survival in patients receiving CABG who were undergoing hemodialysis [33]. In contrast, Hachiro et al. reported that the BITA did not improve the long-term prognosis of patients undergoing dialysis [11]. Further, they described the benefits of the GEA, rather than those of the SVG, as a bypass to the right coronary artery [11]. In 2019, a prospective randomized assignment study reported that the outcomes of using the LITA were not significantly different from those of using the BITA over a 10-year distant survival period. However, the study included a group in which the radial artery was used as a second graft. They also described that multiple artery grafts are superior to single artery grafts, and that further studies are needed [34].

Considering a bias in the preoperative status related to CKD severity regarding the overall survival rate, we confirmed the graft patency rate in patients who underwent coronary CT and CAG in the long-term follow-up. However, most G4 cases lacked contrast studies as they were performed immediately before dialysis; moreover, we could determine the long-term patency rates of the LITA, RITA, and SVG in only a limited number of patients with G5. The RA is almost unavailable in patients undergoing dialysis and patients with severe CKD due to the creation of dialysis shunts. Moreover, the GEA is practically untouchable due to advanced atherosclerosis. However, as arteriosclerosis and stenosis progress in the abdominal artery, common iliac artery, and lower limb artery, the ITA grows as a collateral circulation and often extends continuously to the upper abdominal wall artery. Therefore, using the RITA is beneficial and may facilitate bypassing the right and left coronary artery territories. In our study, long-term follow-up revealed that the RITA had good patency with little occlusion. In contrast, worsening CKD tends to be associated with lower extremity swelling and congestive phlebitis, with the SVG often showing inflammation and intimal thickening. The RITA is a better option than the SVG in cases where the RA or GEA cannot be used (G4–G5, especially for patients undergoing hemodialysis). Kraler et al. reported that the ITA's resilience to atherosclerosis stems from two main biological factors: (1) the ITA's unique anatomy, which promotes positive blood flow independent of age (thereby promoting anti-inflammatory and cytoprotective mechanisms); and (2) the unique biology of the endothelium and vascular smooth muscle cells present in its walls. However, in spite of extensive research on the unique vascular biology of the ITAs, the fundamental mechanisms underlying their extraordinary resilience to atherosclerosis remain unclear. Thus, deciphering ITA atheroresistance may be the key to uncovering new molecular targets to overcome the high residual cardiovascular risk in patients with coronary disease. [35].

## 5. Limitations

This study has some limitations. First, this was a retrospective study, which has an inherent drawback of selection bias; moreover, unmeasured confounding factors may have influenced the results. The limited use of the GEA may also have affected the results. Second, as most patients underwent revascularization using OPCAB at a single facility, the generalizability of our findings to foreign facilities, where cardiopulmonary bypass is more common, is limited. Finally, the number of patients who underwent postoperative cineangiography or coronary 3D-CT examinations was low, especially in the G4 group. Therefore, we could not assess the correlation of the patency rate of BITA grafts with long-term survival rates. The strength of this study is that we were able to offset patients' background regarding chronic kidney disease using the PSM method. In a real-world setting, we were able to analyze the graft patency rate and, therefore, the possibility of graft selection in patients with chronic kidney disease, which may be otherwise challenging.

## 6. Conclusion

Our findings demonstrated that worsening CKD was associated with worsening surgical and long-term outcomes. In-hospital mortality after CABG was not related to renal function, but the severity of CKD strongly influenced long-term outcomes. Along with the severity of renal function, age and operative time were independent risk factors of long-term survival. The BITA grafts did not improve prognosis, but graft patency was superior in the LITA and RITA compared to the patency in the SVG among patients in the G5 group. It is crucial to determine and implement a treatment plan, including graft selection, within a limited time frame in patients with renal dysfunction. Future research is warranted to investigate the role of proteinuria and hypoalbuminemia associated with chronic kidney disease in CABG surgical outcomes and long-term prognosis in such cases.

## Figures and Tables

**Table 1:** Preoperative baseline characteristics of the patients.

Chronic kidney disease (eGFR: mL/min/m^2^)	G1 ≥90	G2 60–89	G3 30–59	G4 15–29	G5 <15	*P* value
Number of patients	128	1696	576	101	282	
eGFR	100.0 ± 18.2	74.3 ± 6.9	47.7 ± 8.3	23.6 ± 4.3	6.0 ± 3.3	<0.001
Hemodialysis	0	0	0	0	256(90.8%)	<0.001
Age (years)	48.0 ± 12.2	67.8 ± 8.9	72.0 ± 8.3	70.1 ± 8.5	65.3 ± 10.4	<0.001
Male	108 (84.4%)	1361 (80.3%)	501 (87.0%)	80 (79.2%)	231 (81.9%)	0.005
BMI (kg/m^2^)	24.8 ± 4.2	23.9 ± 3.2	23.7 ± 3.3	23.7 ± 3.2	23.2 ± 3.8	<0.001
Dyslipidemia	95 (74.2%)	1310 (77.2%)	414 (71.9%)	83 (82.2%)	187 (66.3%)	<0.001
LDL-cholesterol (mg/dL)	109.9 ± 40.9	100.5 ± 31.9	97.9 ± 32.2	97.9 ± 37.4	86.9 ± 32.7	<0.001
Triglyceride (mg/dL)	141.5 ± 88.1	130.5 ± 89.0	133.7 ± 85.9	153.5 ± 82.7	128.1 ± 75.3	0.063
Diabetes mellitus	63 (49.2%)	890 (52.5%)	334 (58.0%)	75 (74.3%)	199 (70.6%)	<0.001
Medication	33 (25.8%)	468 (27.6%)	157 (27.3%)	36 (35.6%)	68 (24.1%)	0.282
Insulin user	20 (15.6%)	223 (13.2%)	114 (19.8%)	32 (31.7%)	90 (31.9%)	<0.001
Hemoglobin A1c (%)	6.3 ± 1.5	6.3 ± 1.2	6.3 ± 1.1	6.4 ± 1.3	6.0 ± 1.1	0.008
Hypertension	64 (50.0%)	1223 (72.2%)	459 (80.0%)	82 (81.2%)	231 (81.9%)	<0.001
Smoker	82 (56.3%)	1048 (62.0%)	360 (62.8%)	65 (64.4%)	168 (59.6%)	0.594
Chronic lung disease	7 (5.5%)	146 (8.6%)	70 (12.2%)	10 (9.9%)	25 (8.9%)	0.067
Peripheral arterial disease	4 (3.1%)	220 (13.0%)	103 (17.9%)	23 (22.8%)	77 (27.3%)	<0.001
History of stroke/TIA	13 (10.1%)	210 (12.4%)	129 (22.4%)	32 (31.7%)	66 (23.4%)	<0.001
Malignancy	9 (7.0%)	195 (11.5%)	95 (16.5%)	16 (15.8%)	41 (14.5%)	0.004
Collagen disease	5 (3.9%)	30 (1.8%)	15 (2.6%)	7 (6.9%)	7 (2.5%)	0.039
Family history of coronary disease	33 (26.0%)	415 (24.6%)	115 (20.0%)	15 (14.9%)	65 (23.1%)	0.039
Previous myocardial infarction	46 (35.9%)	633 (37.3%)	270 (46.9%)	49 (48.5%)	137 (48.6%)	<0.001
NYHA class	15 (4.2%)	46 (3.6%)	35 (4.6%)	7 (6.7%)	17 (6.1%)	0.033
Class III	35 (9.8%)	148 (11.6%)	91 (11.9%)	17 (16.4%)	52 (18.6%)	
Class IV	15 (4.2%)	46 (3.6%)	35 (4.6%)	7 (6.7%)	17 (6.1%)	0.033
Atrial fibrillation	0 (0%)	33 (2.0%)	29 (5.0%)	4 (4.0%)	13 (4.6%)	<0.001
Number of diseased vessels						
1VD	10 (7.8%)	82 (4.8%)	21 (3.7%)	2 (2.0%)	11 (3.9%)	0.178
2VD	23 (18.0%)	267 (15.7%)	84 (14.6%)	18 (17.8%)	49 (17.4%)	0.753
3VD	58 (45.3%)	737 (43.5%)	284 (49.3%)	53 (52.5%)	133 (47.2%)	0.075
Left main trunk	37 (28.9%)	607 (35.8%)	185 (32.1%)	28 (27.7%)	87 (30.9%)	0.094
Poor LVEF (EF < 40%)	12 (9.5%)	119 (7.1%)	92 (16.1%)	16 (15.8%)	68 (24.4%)	<0.001
LVEF (%)	58.7 ± 13.2	58.8 ± 12.4	55.2 ± 14.4	53.1 ± 13.7	49.6 ± 14.1	<0.001
Mitral regurgitation (grade > II)	0 (0%)	33 (2.0%)	20 (3.5%)	10 (9.9%)	15 (5.3%)	<0.001
Preoperative IABP use	5 (3.9%)	66 (3.9%)	23 (4.0%)	7 (6.9%)	14 (5.0%)	0.657
Hemoglobin (g/dL)	13.8 ± 1.6	13.3 ± 1.6	12.5 ± 1.8	11.1 ± 1.6	11.1 ± 1.7	<0.001
C reactive protein (mg/dL)	0.63 ± 1.65	0.49 ± 1.32	0.71 ± 1.64	0.91 ± 1.72	1.03 ± 2.08	<0.001
Bain natriuretic peptide (pg/mL)	63.0 ± 102.8	110.9 ± 196.2	167.5 ± 230.6	300.6 ± 374.9	1019.6 ± 1420.4	<0.001
Japan score	0.93 ± 1.38	1.57 ± 2.55	2.23 ± 4.33	4.98 ± 6.03	7.37 ± 7.98	<0.001
Euroscore II	1.20 ± 1.26	1.88 ± 2.72	3.23 ± 3.17	5.18 ± 5.99	4.11 ± 4.03	<0.001

eGFR: estimated glomerular filtration rate; NYHA: New York Heart Association functional classification; 1VD: one-vessel disease; 2VD: two-vessel disease; 3VD: three-vessel disease; LVEF: left ventricular ejection fraction; IABP: intra-aortic balloon pumping; BMI: body mass index.

**Table 2:** Intraoperative patient characteristics and early results.

Chronic kidney disease (mL/min/m^2^)	G1 ≥90	G2 60–89	G3 30–59	G4 15–29	G5 <15	*P* value
Number of patients	128	1696	576	101	282	
Off-pump CABG	119 (93.0%)	1672 (98.6%)	564 (97.0%)	98 (97.0%)	275 (97.5%)	0.007
No. distal anastomoses	3.4 ± 1.4	3.4 ± 1.3	3.5 ± 1.3	3.6 ± 1.4	3.1 ± 1.3	<0.001
LITA	125 (97.6%)	1661 (97.9%)	565 (98.1%)	98 (97.0%)	274 (97.2%)	0.894
RITA	86 (67.2%)	971 (57.3%)	347 (60.3%)	58 (57.4%)	157 (55.7%)	0.150
Bilateral ITA	84 (66.1%)	955 (56.9%)	339 (59.3%)	56 (56.0%)	152 (54.5%)	0.204
Gastroepiploic artery	51 (39.8%)	603 (35.6%)	217 (37.7%)	40 (39.6%)	62 (22.0%)	<0.001
Radial artery	38 (29.7%)	369 (21.8%)	93 (16.2%)	5 (5.0%)	1 (0.4%)	<0.001
Saphenous vein	30 (23.4%)	742 (43.8%)	296 (51.4%)	54 (53.5%)	148 (52.5%)	<0.001
Operating time(min)	295.3 ± 94.8	283.7 ± 82.5	293.4 ± 82.9	284.5 ± 73.4	265.9 ± 73.1	<0.001
Postoperative IABP	6 (4.7%)	70 (4.1%)	23 (4.0%)	5 (5.0%)	15 (5.3%)	0.901
Reexploration	0 (0%)	11 (0.7%)	4 (0.7%)	0 (0%)	3 (1.1%)	0.458
Respiratory complications	11 (8.6%)	124 (7.3%)	58 (10.1%)	12 (11.9%)	36 (12.8%)	0.018
Postoperative acute renal injury	0 (0%)	27 (2.0%)	33 (6.9%)	29 (31.2%)	279 (98.9%)	<0.001
Cerebrovascular complications	1 (0.8%)	12 (0.7%)	15 (2.6%)	1 (1.0%)	5 (1.8%)	0.016
Any infection	3 (2.3%)	22(1.3%)	13(2.3%)	3(3.0%)	16(5.7%)	0.001
Deep sternal wound infection	2 (1.6%)	6 (0.4%)	2 (0.4%)	1 (1.0%)	2 (0.7%)	0.477
New postoperative atrial fibrillation	12 (9.4%)	423 (24.9%)	186 (32.3%)	29 (28.7%)	78 (27.7%)	<0.001
Blood transfusion	11 (8.6%)	298 (17.6%)	213 (37.0%)	51 (50.5%)	170 (60.3%)	<0.001
Hospital mortality	1 (0.8%)	9 (0.5%)	6 (1.0%)	1 (1.0%)	13 (4.6%)	<0.001
Hospital stay (days)	9.3 ± 3.8	10.4 ± 6.5	13.1 ± 16.2	15.1 ± 14.1	17.9 ± 25.1	<0.001

CABG: coronary artery bypass grafting; LITA: left internal thoracic artery; RITA: right internal thoracic artery; bilateral ITA: bilateral internal thoracic artery; IABP: intra-aortic balloon pumping; BMI: body mass index.

**Table 3:** Multivariate predictors for hospital mortality.

	Univariate		Multivariate	
	OR (95% CI)	*P* value	OR (95% CI)	*P* value
Age (years)	1.03 (0.99–1.07)	0.101	1.03 (0.99–1.08)	0.113
Male sex (male = 1)	0.88 (0.36–2.16)	0.783		
Hypertension (yes = 1)	1.15 (0.49–2.69)	0.742		
Hyperlipidemia (yes = 1)	0.43 (0.21–0.89)	0.031	0.58 (0.27–1.25)	0.162
Diabetes (yes = 1)	0.89 (0.43–1.84)	0.762		
Peripheral arterial disease (yes = 1)	2.39 (1.09–5.26)	0.031	1.25 (0.52–3.02)	0.621
Previous stroke (yes = 1)	3.05 (1.44–6.46)	0.003	2.21 (0.97–5.02)	0.061
CKD stage (reference = G1)				
G2	0.68 (0.09–5.39)	0.712	0.34 (0.04–3.07)	0.343
G3	1.34 (0.16–11.20)	0.791	0.42 (0.04–4.19)	0.462
G4	1.27 (0.08–20.56)	0.871	0.32 (0.02–6.08)	0.444
G5	6.14 (0.79–47.43)	0.084	1.69 (0.19–14.59)	0.643
MR ≥ moderate (yes = 1)	5.56 (1.89–16.34)	0.001	2.78 (0.84–9.20)	0.092
Preoperative AF (yes = 1)	2.48 (0.58–10.61)	0.224		
LMT and/or 3VD (yes = 1)	0.85 (0.36–2.00)	0.712		
LVEF <40% (yes = 1)	6.39 (3.05–13.18)	<0.001	4.36 (1.94–9.79)	<0.001
BITA (yes = 1)	0.85 (0.56–2.48)	0.671		
SVG (yes = 1)	0.91 (0.44–1.88)	0.802		
GEA (yes = 1)	0.46 (0.19–1.13)	0.094	1.97 (0.76–5.09)	0.163
RA (yes = 1)	0.90 (0.34–2.36)	0.832		
LAA amputation (yes = 1)	0.93 (0.42–2.04)	0.851		

PCI: percutaneous coronary intervention; CKD: chronic kidney disease; MR: mitral regurgitation; AF: atrial fibrillation; LMT: left main trunk; 3VD: three-vessel disease; LVEF: left ventricular ejection fraction; BITA: bilateral internal thoracic artery; SVG: saphenous vein graft; GEA: gastroepiploic artery; RA: radial artery; LAA amputation: left atrial appendage amputation.

**Table 4:** Multivariate predictors for mortality in the follow up.

	Univariate		Multivariate	
	OR (95% CI)	*P* value	OR (95% CI)	*P* value
Age (years)		<0.001	1.05 (1.04–1.06)	<0.001
Male sex (male = 1)	1.19 (0.97–1.46)	0.091		
Hypertension (yes = 1)	1.20 (1.01–1.43)	0.042	1.02 (0.85–1.22)	0.853
Hyperlipidemia (yes = 1)	0.62 (0.53–0.72)	<0.001	0.72 (0.61–0.85)	<0.001
Diabetes (yes = 1)	1.31 (1.13–1.53)	<0.001	1.26 (1.07–1.47)	0.004
Peripheral arterial disease (yes = 1)	2.07 (1.73–2.49)	<0.001	1.59 (1.32–1.93)	<0.001
Previous stroke (yes = 1)	1.85 (1.55–2.21)	<0.001	1.25 (1.04–1.51)	0.025
CKD stage (reference = G1)				
G2	3.55 (1.76–7.16)	<0.001	1.30 (0.63–2.68)	0.472
G3	7.52 (3.71–15.25)	<0.001	1.95 (0.94–4.06)	0.073
G4	9.73 (4.51–21.02)	<0.001	2.82 (1.27–6.24)	0.012
G5	16.36 (7.96–33.63)	<0.001	5.78 (2.76–12.09)	<0.001
MR ≥ moderate (yes = 1)	2.38 (1.64–3.46)	<0.001	1.65 (1.13–2.42)	0.011
Preoperative AF (yes = 1)	2.17 (1.52–3.10)	<0.001	1.65 (1.14–2.38)	0.010
LMT and/or 3VD (yes = 1)	1.23 (1.02–1.49)	0.032	1.28 (1.04–1.57)	0.022
LVEF <40% (yes = 1)	1.82 (1.48–2.24)	<0.001	1.75 (1.41–2.18)	<0.001
BITA (yes = 1)	0.77 (0.66–0.90)	<0.001	0.76 (0.65–0.90)	<0.001
SVG (yes = 1)	1.33 (1.14–1.54)	<0.001	1.03 (0.88–1.22)	0.702
GEA (yes = 1)	0.81 (0.69–0.94)	0.008	1.07 (0.89–1.28)	0.463
RA (yes = 1)	0.84 (0.70–1.01)	0.072		
LAA amputation (yes = 1)	0.74 (0.57–0.94)	0.023	0.71 (0.55–0.92)	0.012

PCI: percutaneous coronary intervention; CKD: chronic kidney disease; MR: mitral regurgitation; AF: atrial fibrillation; LMT: left main trunk; 3VD: three-vessel disease; LVEF: left ventricular ejection fraction; BITA: bilateral internal thoracic artery; SVG: saphenous vein graft; GEA: gastroepiploic artery; RA: radial artery; LAA amputation: left atrial appendage amputation.

**Table 5:** Patients' baseline characteristics.

	G1–2	G3–5	
Chronic kidney disease (eGFR: mL/min/m^2^)	≥60	<59	*P* value
Number of patients	909	909	
Male	664 (73.0%)	771 (84.8%)	<0.001
Age (years)	60.1 ± 9.7	66.9 ± 9.3	0.862
Male	108 (84.4%)	1361 (80.3%)	<0.001
Diabetes mellitus	448 (53.7%)	516 (56.8%)	0.187
Diabetes mellitus (medication)	224 (24.6%)	259 (28.5%)	0.301
Hyperlipidemia	695 (76.5%)	676 (74.4%)	0.301
Hypertension	666 (73.3%)	682 (75.0%)	0.391
History of stroke/TIA	209 (23.0%)	132 (14.5%)	<0.001
Peripheral arterial disease	166(18.3%)	1(14.2%)	0.019
Chronic lung disease	60 (6.6%)	95 (10.5%)	0.003
Previous myocardial infarction	384 (42.2%)	408 (44.9%)	0.256
Previous PCI	168 (18.5%)	215 (23.7%)	0.007
Mitral regurgitation (grade > II)	68 (7.5%)	21 (2.3%)	<0.001
Emergency	80 (8.8%)	61(6.7%)	0.096
Number of diseased vessels	3.11 ± 0.85	3.09 ± 0.83	0.084
LVEF (%)	56.6 ± 13.5	57.2 ± 13.3	0.506
Number of bypasses	3.24 ± 1.32	3.41 ± 1.24	0.195
Operative time (min)	267.1 ± 70.0	279.6 ± 72.7	0.401
Duration of follow-up (years)	8.7 ± 5.0	6.0 ± 4.3	<0.001

eGFR: estimated glomerular filtration rate; PCI: percutaneous coronary intervention; LVEF: left ventricular ejection fraction.

**Table 6:** Multivariate predictors of mortality in the follow-up period.

	Univariate		Multivariate	
	OR (95% CI)	*P* value	OR (95% CI)	*P* value
Sex (male)	0.674 (0.370–1.226)	0.196	0.887 (0.789–0.999)	0.047
Age	0.984 (0.963–1.007)	0.169	1.018 (1.013–1.023)	<0.001
Diabetes mellitus	0.510 (0.237–1.096)	0.084	0.959 (0.872–1.055)	0.391
Hyperlipidemia	1.207 (0.726–2.007)	0.468		
Hypertension	0.825 (0.479–1.419)	0.486		
History of stroke/TIA	1.196 (0.644–2.222)	0.571		
Peripheral arterial disease	1.421 (0.714–2.827)	0.317		
Chronic lung disease	0.985 (0.441–2.203)	0.972		
Previous myocardial infarction	1.011 (0.643–1.591)	0.962		
Previous PCI	0.984 (0.569–1.701)	0.954		
Mitral regurgitation (grade > II)	2.079 (0.486–8.895)	0.324		
Emergency	1.962 (0.694–5.545)	0.204		
Number of diseased vessels	0.926 (0.708–1.211)	0.572		
LVEF	1.008 (0.990–1.026)	0.391		
Number of bypasses	0.860 (0.682–1.086)	0.205		
Operative time	1.009 (1.004–1.013)	<0.001	1.023(1.021–1.024)	<0.001

PCI: percutaneous coronary intervention; LVEF: left ventricular ejection fraction.

**Table:** (a) Graft patency rate within 1 postoperative year

		LITA					RITA		
Findings	Patent	String/stenosis	Occlusion	Number	Findings	Patent	String/stenosis	Occlusion	Number
G1	81 (98.8%)	0	1 (1.2%)	82	G1	58 (92.1%)	3 (4.7%)	2 (3.2%)	63
G2	314 (97.5%)	4 (1.2%)	4 (1.3%)	322	G2	200 (93.5%)	12 (5.6%)	2 (0.9%)	214
G3	139 (96.5%)	5 (3.5%)	0	144	G3	88 (93.6%)	3 (3.2%)	3 (3.2%)	94
G4	2 (100%)	0	0	2	G4	0	0	0	0
G5	60 (96.7%)	2 (3.3%)	0	62	G5	27 (100%)	0	0	27
		GEA					SVG		
Findings	Patent	String/stenosis	Occlusion	Number	Findings	Patent	String/stenosis	Occlusion	Number
G1	26 (96.3%)	0	1 (3.7%)	27	G1	22 (84.6%)	1 (3.9%)	3 (11.5%)	26
G2	95 (88.8%)	2 (1.9%)	10 (9.3%)	107	G2	149 (94.9%)	1 (0.6%)	7 (4.5%)	157
G3	44 (93.6%)	1 (2.1%)	2 (4.3%)	47	G3	72 (97.2%)	1 (1.4%)	1 (1.4%)	74
G4	0	0	0	0	G4	1 (50.0%)	0	1 (50.0%)	2
G5	13 (100%)	0	0	13	G5	32 (88.9%)	1 (2.8%)	3 (8.3%)	36

LITA: left internal thoracic artery; RITA: right internal thoracic artery; GEA: gastroepiploic artery; SVG: saphenous vein graft; Number: number of observed patients.

**Table:** (b) Graft patency rate between postoperative years 1 and 5

		LITA					RITA		
Findings	Patent	String/stenosis	Occlusion	Number	Findings	Patent	String/stenosis	Occlusion	Number
G1	52 (98.1%)	1 (1.9%)	0	53	G1	35 (87.5%)	3 (7.5%)	2 (5.0%)	40
G2	164 (94.8%)	2 (1.2%)	7 (4.0%)	173	G2	102 (90.3%)	5 (4.4%)	6 (5.3%)	113
G3	51 (98.1%)	0	1 (1.9%)	52	G3	28 (87.5%)	2 (6.3%)	2 (6.2%)	32
G4	3 (100%)	0	0	3	G4	0	0	0	0
G5	27 (100%)	0	0	27	G5	18 (100%)	0	0	18
		GEA					SVG		
Findings	Patent	String/stenosis	Occlusion	Number	Findings	Patent	String/stenosis	Occlusion	Number
G1	15 (93.8%)	0	1 (6.2%)	16	G1	17 (85.0%)	0	3 (15.0%)	20
G2	57 (89.1%)	0	7 (10.9%)	64	G2	67 (80.7%)	1 (1.2%)	15 (18.1%)	83
G3	11 (84.6%)	1 (7.7%)	1 (7.7%)	13	G3	26 (89.7%)	0	3 (10.3%)	29
G4	2 (100%)	0	0	2	G4	1 (100%)	0	0	1
G5	5 (100%)	0	0	5	G5	12 (75.0%)	1 (6.3%)	3 (18.7%)	16

LITA: left internal thoracic artery; RITA: right internal thoracic artery; GEA: gastroepiploic artery; SVG: saphenous vein graft; Number: number of observed patients.

**Table:** (c) Graft patency rate between 5 and 10 years postoperatively

		LITA					RITA		
Findings	Patent	String/stenosis	Occlusion	Number	Findings	Patent	String/stenosis	Occlusion	Number
G1	35 (92.1%)	0	3 (7.9%)	38	G1	18 (85.7%)	1 (4.8%)	2 (9.5%)	21
G2	113 (95.0%)	3 (2.5%)	3 (2.5%)	119	G2	57 (87.7%)	2 (3.1%)	6 (9.2%)	65
G3	39 (95.1%)	0	2 (4.9%)	41	G3	16 (84.2%)	2 (10.5%)	1 (5.3%)	19
G4	3 (100%)	0	0	3	G4	3 (100%)	0	0	3
G5	14 (93.3%)	1 (6.7%)	0	15	G5	7 (100%)	0	0	7
		GEA					SVG		
Findings	Patent	String/stenosis	Occlusion	Number	Findings	Patent	String/stenosis	Occlusion	Number
G1	13 (92.9%)	1 (7.1%)	0	14	G1	14 (82.4%)	0	3 (17.6%)	17
G2	44 (81.5%)	1 (1.9%)	9 (16.6%)	54	G2	45 (83.3%)	1 (1.9%)	8 (14.8%)	54
G3	12 (80.0%)	1 (6.7%)	2 (13.3%)	15	G3	13 (81.3%)	0	3 (18.7)	16
G4	2 (66.7%)	0	1 (33.3%)	3	G4	1 (100%)	0	0	1
G5	3 (100%)	0	0	3	G5	4 (57.1%)	0	3 (42.9%)	7

LITA: left internal thoracic artery; RITA: right internal thoracic artery; GEA: gastroepiploic artery; SVG: saphenous vein graft; Number: number of observed patients.

## Data Availability

The data used to support the findings of this study are available from the corresponding author upon request.
